# Exploring Pembrolizumab-Induced Myocarditis, Myositis, and Myasthenia Gravis: A Comprehensive Literature Review and Case Presentation on Bladder Cancer

**DOI:** 10.7759/cureus.49867

**Published:** 2023-12-03

**Authors:** Divya Shah, Kristen Young

**Affiliations:** 1 Internal Medicine, University of Arizona College of Medicine - Phoenix, Phoenix, USA; 2 Rheumatology, University of Arizona College of Medicine - Phoenix, Phoenix, USA

**Keywords:** pembrolizumab-induced myasthenia gravis, immune therapy-mediated myocarditis, myositis-mg overlap, pembrolizumab side effect, new drug reaction

## Abstract

Pembrolizumab, a humanized monoclonal anti-programmed cell death protein 1 (PD1) antibody, has shown efficacy in various malignancies. This article presents a case of stage III squamous cell carcinoma of the bladder treated with pembrolizumab, resulting in the development of a rare overlap syndrome known as myocarditis, myositis, and myasthenia gravis (IM3OS). While immune checkpoint inhibitors like pembrolizumab demonstrate notable antitumor activity, they also pose the risk of severe immune-related adverse events. The case underscores the importance of the early detection of IM3OS and the potential dangers associated with delayed diagnosis, offering valuable insights for healthcare providers managing patients on pembrolizumab therapy.

## Introduction

Pembrolizumab, an immune checkpoint inhibitor, has revolutionized cancer treatment since its Food and Drug Administration (FDA) approval in 2014 for advanced melanoma [1]. Its success extends to various oncologic conditions, including squamous cell carcinoma of the bladder. By targeting programmed cell death protein 1 (PD1) on T-cells, pembrolizumab stimulates the immune system to combat cancer cells. Despite its promising antitumor effects, immune checkpoint inhibitors may lead to rare and severe immune-related adverse effects (irAEs) affecting various organs [2]. As pembrolizumab use becomes increasingly prevalent, a deeper understanding of its benefits and potential adverse events is crucial for the medical community.

The article introduces a specific overlap syndrome known as IM3OS, involving the simultaneous occurrence of myocarditis, myositis, and myasthenia gravis. This syndrome is characterized by an abrupt onset following drug initiation (often a single dose), rapid clinical progression, and a grim prognosis [3]. Patients with immune-related myasthenia gravis or myositis who also exhibit myocarditis and elevated serum troponin face a particularly poor clinical prognosis. IM3OS is associated with a high mortality rate, primarily attributed to complications such as cardiac arrhythmias and neuromuscular issues leading to respiratory failure [3]. While immune-related adverse events of immune checkpoint inhibitors are documented, the article highlights the rarity of pembrolizumab-induced myocarditis with the simultaneous involvement of multiple organs, including respiratory and skeletal muscles.

This case was presented as a poster at the 2022 Congress of Clinical Rheumatology-West Meeting on October 20, 2022.

## Case presentation

We present a 74-year-old male with a medical history of hypertension, hyperlipidemia, and a diagnosis of stage III squamous cell carcinoma of the bladder six months ago. Rather than pursuing chemotherapy, he opted for a programmed death-ligand 1 (PD-L1) test, indicating a PD-L1 expression exceeding 76%. Consequently, he initiated immune checkpoint inhibitor therapy with pembrolizumab. A week into the treatment, he presented at a regional hospital, reporting bilateral proximal muscle pain, weakness, and progressive bilateral ptosis and ophthalmoplegia. Rhabdomyolysis was diagnosed due to markedly elevated creatine kinase (CK) levels (>8000 u/L), and he was found to have a complete heart block, leading to the insertion of a permanent pacemaker. On the tenth day, the patient was transferred to a different facility due to unresponsive myositis and ptosis. The patient presented with enduring and intense muscle pain, notably in the posterior calves, legs, and shoulders, along with weakness and involvement of cranial nerves III, IV, and VI during the physical examination. The patient did not report experiencing painful vision, skin rashes, dyspnea, diarrhea, abdominal pain, or joint swelling. Laboratory results indicated elevated creatine kinase-MB (CK-MB), troponin, N-terminal pro-brain natriuretic peptide (NT-proBNP), aspartate aminotransferase (AST), alanine aminotransferase (ALT), and creatinine (Table 1). Imaging studies ruled out thymoma-induced myasthenia gravis but revealed features suggestive of myocarditis on cardiac MRI.

**Table 1:** Key laboratory findings upon the patient's initial presentation u: units; L: liter; ng: nanogram; pg: picogram; mL: milliliter; dL: deciliter; min: minute; NT-proBNP: N-terminal pro-brain natriuretic peptide; AST: aspartate aminotransferase; ALT: alanine aminotransferase; eGFR: estimated glomerular filtration rate

Lab name	Lab value	Reference range
CK-MB	>6000 u/L	0-25 u/L
Troponin	11.9 ng/mL	<0.03 ng/mL
NT-proBNP	>1800 pg/mL	<300 pg/mL
AST	651 u/L	0-40 u/L
ALT	271 u/L	0-40 u/L
eGFR	59 mL/min	>90 mL/min
Creatinine	2.86 mg/dL	0.7-1.25 mg/dL

**Figure 1:**
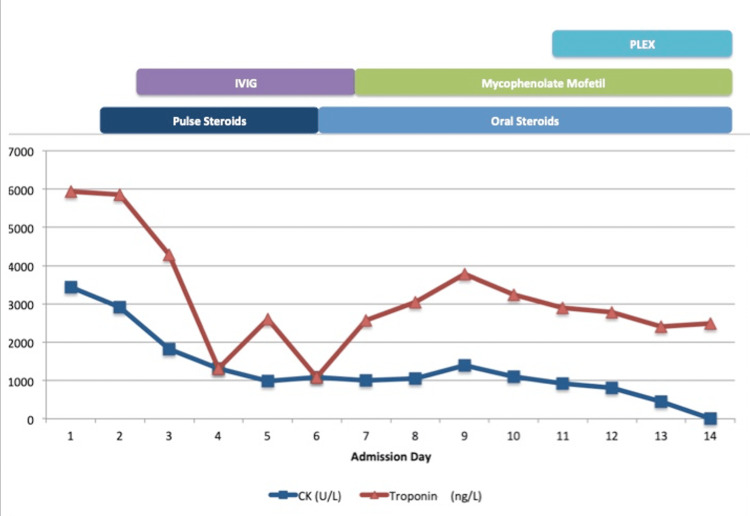
Key cardiac markers and treatment timeline CK: creatinine kinase

The patient, exhibiting sinus rhythm on the electrocardiogram, presented echocardiographic abnormalities, including apical and inferior wall motion abnormalities and a depressed ejection fraction of 40%. Even though acetylcholine receptor antibodies and voltage-gated calcium channel antibodies tested negative, the detection of anti-striated muscle antibodies prompted treatment involving intravenous immunoglobulin, high-dose glucocorticoids (one gram for five days), and mycophenolate mofetil. However, the patient’s symptoms showed minimal improvement, prompting the initiation of plasma exchange (Figure 1). Despite aggressive interventions, the patient developed refractory hypotension, experienced pulseless electrical activity, and succumbed to the condition on hospital day 14. The diagnosis in this case was the overlap syndrome known as IM3OS, emphasizing the challenges associated with managing this rare condition associated with pembrolizumab.

## Discussion

The passage underscores the increasing use of pembrolizumab and the critical necessity for clinicians to anticipate and identify potential side effects, particularly the rare pembrolizumab-associated overlap syndrome, IM3OS. To date, immune checkpoint inhibitors, such as nivolumab (with or without ipilimumab), cemiplimab, atezolizumab, avelumab, and durvalumab, have been implicated in patients experiencing myocarditis, often manifesting as arrhythmias, along with myositis and/or myasthenia gravis [3]. Despite limited knowledge of this syndrome, a comprehensive search on PubMed revealed a single case implicating pembrolizumab in IM3OS development [4]. Early intervention with steroids, in this case, yielded favorable outcomes for neuromuscular complications. This case and the one detailed in this article underscore the importance of early detection of IM3OS and emphasize the dangers linked with delayed diagnosis. Unlike many published cases that primarily focus on immune checkpoint inhibitors’ cardiovascular toxicity or peripheral nervous system effects, this article explicitly highlights pembrolizumab-induced myocarditis and its associated immune-related adverse events like myositis and myasthenia gravis within the context of the overlap syndrome. The goal of this study is to provide valuable insights to healthcare professionals for improved clinical practice.

The presented case is considered one of the few instances of pembrolizumab-associated IM3OS. The unique aspect of interest lies in the observed mechanism, suggesting that myocarditis might be less responsive to immunosuppressive treatments compared to myositis, potentially contributing to a fatal outcome [5]. The mechanism of cardiovascular immune-related adverse events due to immune checkpoint inhibitors remains poorly understood, with various interconnected theories proposed. These include a potential shared epitope between the tumor and myocardium, leading to cross-reactivity and reactions to self through the activation of preexisting T-cell clones or autoantibodies targeting troponin [6]. Other considerations involve the release of intracellular antigens upon tumor necrosis, T-cell infiltration due to overexpression of PD1 on cardiomyocytes, or the release of proinflammatory cytokines [6]. The passage emphasizes the need for further research to elucidate the underlying mechanisms of this association.

For future instances with pembrolizumab, the significance of regular screening for IM3OS using cardiac cytolysis biomarkers (such as CK-MB and troponin I) to enable early detection is underscored. Additionally, routine electrocardiograms and echocardiography are suggested to prevent lethal cardiac damage through timely intervention. A comprehensive management approach is deemed essential for early detection and intervention in IM3OS cases, and further studies are encouraged to refine cancer management strategies involving immune checkpoint inhibitors.

## Conclusions

This article highlights the importance of diagnosing and managing IM3OS, particularly myocarditis, and advocates for the development of biomarkers to predict its occurrence and severity. Providers offering anticipatory guidance should be aware of this potential side effect, prompting clinicians to include ICIs like pembrolizumab in their differential diagnoses for unexplained concurrent myocarditis, myositis, and myasthenia gravis. Early recognition of this triad and prompt management involving steroids and supportive care through interdisciplinary medical care may enhance clinical outcomes. The case discussed is noted as one of the initial reports of pembrolizumab-associated IM3OS, emphasizing the need for ongoing monitoring to determine the prevalence of this syndrome in other cases.

## References

[1] Raedler LA (2015). Keytruda (pembrolizumab): first PD-1 inhibitor approved for previously treated unresectable or metastatic melanoma. Am Health Drug Benefits.

[2] Baxi S, Yang A, Gennarelli RL, Khan N, Wang Z, Boyce L, Korenstein D (2018). Immune-related adverse events for anti-PD-1 and anti-PD-L1 drugs: systematic review and meta-analysis. BMJ.

[3] Pathak R, Katel A, Massarelli E, Villaflor VM, Sun V, Salgia R (2021). Immune checkpoint inhibitor-induced myocarditis with myositis/myasthenia gravis overlap syndrome: a systematic review of cases. Oncologist.

[4] Todo M, Kaneko G, Shirotake S (2020). Pembrolizumab-induced myasthenia gravis with myositis and presumable myocarditis in a patient with bladder cancer. IJU Case Rep.

[5] Prevel R, Colin G, Calès V, Renault PA, Mazieres J (2020). Third degree atrio-ventricular blockade during a myocarditis occurring under anti-PD1 : case report and literature review [Article in French]. Rev Med Interne.

[6] Raschi E, Rossi S, De Giglio A, Fusaroli M, Burgazzi F, Rinaldi R, Potena L (2023). Cardiovascular toxicity of immune checkpoint inhibitors: a guide for clinicians. Drug Saf.

